# A simple dummy liver assist device prolongs anhepatic survival in a porcine model of total hepatectomy by slight hypothermia

**DOI:** 10.1186/1471-230X-11-79

**Published:** 2011-07-14

**Authors:** Karolin Thiel, Martin Schenk, Alexander Etspüler, Thomas Schenk, Matthias H Morgalla, Alfred Königsrainer, Christian Thiel

**Affiliations:** 1 Department of General, Visceral and Transplant Surgery, Tuebingen University Hospital, Hoppe-Seyler-Strasse 3, Tuebingen 72076, Germany; 2 Department of Anaesthesiology, Tuebingen University Hospital, Hoppe-Seyler-Strasse 3, Tuebingen 72076, Germany; 3 Department of Neurosurgery, Tuebingen University Hospital, Hoppe-Seyler-Strasse 3, Tuebingen 72076, Germany

## Abstract

**Background:**

Advances in intensive care support such as therapeutic hypothermia or new liver assist devices have been the mainstay of treatment attempting to bridge the gap from acute liver failure to liver transplantation, but the efficacy of the available devices in reducing mortality has been questioned. To address this issue, the present animal study was aimed to analyze the pure clinical effects of a simple extracorporeal dummy device in an anhepatic porcine model of acute liver failure.

**Methods:**

Total hepatectomy was performed in ten female pigs followed by standardized intensive care support until death. Five animals (dummy group, n = 5) underwent additional cyclic connection to an extracorporeal dummy device which consisted of a plasma separation unit. The separated undetoxified plasma was completely returned to the pigs circulation without any plasma substitution or exchange in contrast to animals receiving intensive care support alone (control group, n = 5). All physiological parameters such as vital and ventilation parameters were monitored electronically; laboratory values and endotoxin levels were measured every 8 hours.

**Results:**

Survival of the dummy device group was 74 ± 6 hours in contrast to 53 ± 5 hours of the control group which was statistically significant (p < 0.05). Body temperature 24 hours after hepatectomy was significantly lower (36.5 ± 0.5°C vs. 38.2 ± 0.7°C) in the dummy device group. Significant lower values were measured for blood lactate (1.9 ± 0.2 vs. 2.5 ± 0.5 mM/L) from 16 hours, creatinine (1.5 ± 0.2 vs. 2.0 ± 0.3 mg/dL) from 40 hours and ammonia (273 ± 122 vs. 1345 ± 700 μg/dL) from 48 hours after hepatectomy until death. A significant rise of endotoxin levels indicated the onset of sepsis at time of death in 60% (3/5) of the dummy device group animals surviving beyond 60 hours from hepatectomy.

**Conclusions:**

Episodes of slight hypothermia induced by cyclic connection to the extracorporeal dummy device produced a significant survival benefit of more than 20 hours through organ protection and hemodynamic stabilisation. Animal studies which focus on a survival benefit generated by liver assist devices should especially address the aspect of slight transient hypothermia by extracorporeal cooling.

## Background

Acute liver failure (ALF) is defined as rapid and progressive development of severe acute liver injury with impaired liver synthetic function without a previous history of liver disease. Worsening encephalopathy and cerebral edema escalating in brain stem herniation [1] as well as infection and inflammation with development of systemic inflammatory response syndrome (SIRS) frequently contributing to multiple-organ failure in this setting [2] are the main courses of death. The complexity of metabolic abnormalities resulting from ALF is still incompletely understood hence morbidity and mortality among patients with ALF without liver transplantation remains as high as 85% [3].

Animal models of ALF remain the mainstay of research in development of more efficient artificial [4,5] or bioartificial [6,7] liver assist devices as well as preclinical trials in newly developed therapeutic approaches. Animal models of ALF are mainly based on surgical techniques such as devascularisation [8], ischemia [9], extensive/total liver resection [10,11] or medical interventions such as hepatic intoxication with acetaminophen [12], amanitin [13] or galactosamine [14]. Unfortunately all aforementioned models involve various limitations, thus affecting morbidity in the assessment of a given intervention. Although anhepatic models have been criticized for their irreversibility and lack of circulating products of cell necrosis or inflammatory mediators through the native liver, it is commonly considered to serve as a suitable in vivo model for testing the effectiveness of liver assist devices due to high reproducibility and the total absence of remaining functional liver parenchyma [15-17]. But surprisingly the hereby demonstrated survival benefit of the already established devices could not be reproduced in clinical practise. Considering this fact we decided to revaluate a simple dummy device to verify if experimentally demonstrated survival benefits are properly controlled for side-effects.

Preliminary pigs studies have been carried out to establish a highly reproducible model of total hepatectomy [11] delivering long-term survival under standardized intensive care conditions [18]. It has been demonstrated experimentally and clinically that moderate hypothermia is organ protective in specific life threatening conditions such as cardiac arrest [19], traumatic brain injury [20] or ALF [21]. It is also well known that the use of extracorporeal circuits like hemofiltration, plasmapheresis or liver assist devices result in moderate peripheral cooling of the connected person. Although recent ALF animal models have certainly been controlled for hypothermia [22], various animal studies evaluating liver assist devices or other therapeutical interventions did not report the exact courses of body temperature while connection to the devices [15,23] or did not even test their extracorporeal devices against a dummy device group [23,24]. Assuming that these studies might not be properly controlled for extracorporeal device associated transient hypothermia, we performed the present study to analyze a dummy device effects on hemodynamics, body temperature and survival.

## Methods

### Animal model of ALF

After approval by the institutional review board for animal experiments, ten female German Landrace pigs weighing 36 ± 4 kg underwent total hepatectomy following frontoparietal trepanation for intracranial pressure (ICP) monitoring. All experiments were performed according to the international principles governing research on animals and under the supervision of a veterinarian, who set the guidelines for minimizing pigs suffering.

### Anesthesia

Intramuscular premedication was administered using atropine 0.1% (0.05 mg/kg), ketamine (7 mg/kg), azaperone (10 mg/kg) and diazepam (1 mg/kg). A core body temperature of 38 ± 0.5°C measured by a rectal probe was aimed by using warming blankets throughout the experiment. A stomach tube (Argyle™, Tyco Healthcare, Tullamore, Ireland) was placed for intestinal drainage. After oral intubation with a cuffed endotracheal tube (Lo-Contour™ Magill, Mallinckrodt Medical, Athlone, Ireland) the pigs were ventilated with pressure-controlled ventilation modus (Galileo Gold, Hamilton Medical, Rhaezuens, Switzerland). Arterial blood gas analysis (ABL 625, Radiometer Copenhagen, Denmark) including blood lactate measurement was performed hourly and ventilation was adjusted accordingly. Continuous infusion of ketamine (15 mg/kg/h), fentanyl (0.02 mg/kg/h) and midazolam (0.9 mg/kg/h) was administered to maintain deep anesthesia throughout the experiment. Character of respiration, heart rate, eye movement and pain stimulus was used to confirm depth of anesthesia; if any of these parameters indicated a lessening of anesthesia, infusion rates of anaesthetic agents were increased.

### Surgical procedure and randomization

Animals were kept under standard laboratory conditions and fasted overnight before surgery. They received 2 g ceftriaxon (Rocephin^® ^, Hoffmann-La Roche, Basel, Switzerland) prior to surgery. The superior vena cava through the jugular veins (Multi-Lumen Central Venous Catheter, Arrow International, Reading, PA, USA) and the internal carotid artery (Leadercath, Vygon, Écouen, France) were instrumented to measure mean arterial pressure (MAP) and central venous pressure. Following frontoparietal cranial trepanation, a probe was inserted in the frontal brain parenchyma to measure ICP (Camino^® ^MPM-1 monitor, Integra Neurosciences, Plainsboro, NJ, USA). The abdominal cavity was entered through a midline incision and a urinary catheter (Gentle-Flo™, Tyco Healthcare, Tullamore, Ireland) was placed by cystostomy. Total hepatectomy was performed according to a recently published technique [11] with a Y-graft vascular prosthesis (Uni-Graft^® ^K DV, ITV, Denkendorf, Germany) interposition. Intraoperative blood loss was substituted with porcine erythrocyte and fresh-frozen plasma units. After stabilisation of the hemodynamic situation pigs were randomized in two groups receiving standardized intensive care therapy alone (n = 5) or additional periodic connection to the extracorporeal dummy device system (n = 5).

### Extracorporeal circuit of the dummy device

Cyclic connection of the pigs to the extracorporeal dummy device system started approximately twelve hours after hepatectomy for a time period of twelve hours. This connection cycles were continued every twelve hours for twelve hours. To simulate the experimental setting of an artificial or bioartificial liver assist device, pigs underwent plasma separation (TPE, Prismaflex, Gambro, Hechingen, Germany) with a mean blood flow rate of 120 mL/min and a plasma separation rate of 20 mL/min using a plasma filter (TPE 2000, Gambro, Hechingen, Germany) with a maximal pore size of 0.5 μm. The total extracorporeal volume of the device system was 125 mL ± 10%. The device system including the plasma filter was washed and primed according to the manufacturer's instruction. Heparin (250 U/h) was administered as necessary to avoid clotting in the extracorporeal circuit of the dummy device. The separated plasma fraction was completely returned to the animal via the implanted central vein catheter without any further plasma filtration/detoxification or plasma exchange/replacement.

### Preparation of donor fresh-frozen plasma and erythrocyte units

Blood was collected in blood bag systems (500 mL, Compoflex^® ^Fresenius HemoCare, Bad Homburg, Germany) and centrifuged at 2,500 g for 20 minutes (Heraeus Cryofuge 5500i, Thermo Electron Corporation, Langenselbold, Germany). Plasma fraction was pressed into separate bags and shock-frozen at minus -80°C. Erythrocytes were conserved with 100 mL SAG-Mannitol and stored at 4°C.

### Standardized intensive care therapy

Animals remained in general anesthesia receiving pressure-controlled ventilation until conclusion of the study protocol (15-30 breaths/minute, tidal volume 6-12 mL/kg and oxygen concentration 0.3-1.0, depending on oxygenation). Monitoring throughout the experiment included electrocardiogram, ICP, MAP, central venous pressure, oxygen saturation and core body temperature. Urinary output, haemoglobin, lactate, serum electrolytes, acid-base balance, blood gase analysis and blood glucose levels were monitored hourly and immediately corrected as required. The selected laboratory parameters like prothrombin time, albumin, plasma protein, creatinine, bilirubin and ammonia were measured before, after and every eight hours after hepatectomy until death. All blood samples were obtained from the arterial carotid catheter. Norepinephrine, in combination with fresh-frozen plasma, hydroxyethylstarch 6% (Voluven^® ^HES 130/0.4, Fresenius, Bad Homburg, Germany) and sodium chloride solution 0.9% were used to ensure hemodynamic stability. Algorithms of fluid management, ventilation and intensive care medication have already been reported in detail [18]. Blood glucose levels were maintained > 100 mg/dL with glucose 20% solution. Packed erythrocyte units were given if haemoglobin levels trend to decline below 6 g/dL. To prevent spontaneous bleeding eight fresh-frozen plasma units were given within 24 hours. Pigs received furosemide (maximum 1,000 mg/d) to maintain diuresis as long as possible. Antibiotic prophylaxis with 2 g ceftriaxon was continued daily. Death was defined as a decline of MAP below 30 mmHg under maximal vasopressor support.

### Laboratory analysis

All biochemical parameters as prothrombin time, creatinine, albumin, bilirubin, ammonia, total plasma protein and blood count were measured by the certified laboratories of the University Hospital Tuebingen (Zentrallabor, Innere Medizin IV, University Hospital Tuebingen, Germany). Sample analysis was conducted within 1 hour of collection at each time point.

Endotoxin was measured by a Limulus-Amoebozyte-Lysate assay (Charles River Endosafe, Charleston, SC, USA) and performed according to the manufacturer's instruction. In brief, all endotoxin samples were obtained from the arterial catheter and collected in sterile pyrogen-free vacuum tubes (Endo Tube ET, Chromogenics AB, Moelndal, Sweden). Subsequently probes were centrifuged at 3,000 g for 10 minutes. The supernatant was immediately transferred into biopur-grade reaction tubes (Eppendorf AG, Hamburg, Germany) and stored at -80°C.

After thawing, samples were heat inactivated at 80°C for 10 minutes. Samples underwent cooling for 1 minute at 0°C and ultrasonic bathing for 3 minutes followed by centrifugation at 10,000 g for 60 minutes at 4°C. Supernatants were transferred in micro plates (Microtest 96, Becton Dickinson, Franklin Lakes, NJ, USA) and measured kinetically with the (Endosafe^® ^Endochrome-K™, Charles River Endosafe, Charleston, SC, USA). Spike and recovery assays were performed.

### Statistical Analysis

Mean values of the selected variables determined before, during and after hepatectomy were compared by *t*-Test, (JMP^® ^4.0, SAS Institute, Cary, NC, USA). A p value < 0.05 was considered significant. Results are reported as mean ± standard deviation (SD). Figures are given as mean ± standard error of mean (SEM) of a minimum of two animals per study group.

## Results

### Survival

**Figure 1:**
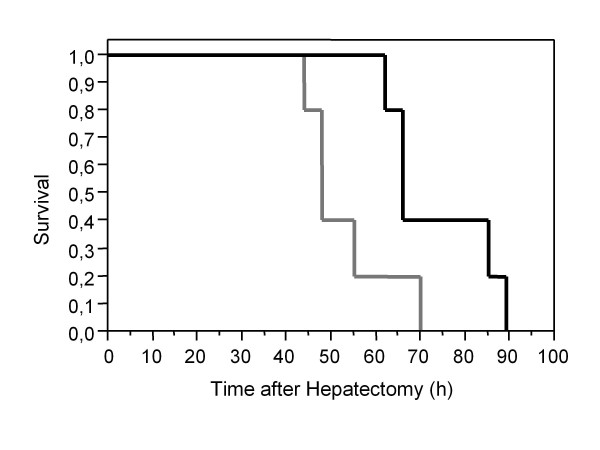
**Kaplan-Meier survival plot for control and dummy device group**. The control group as demonstrated by the grey line survived 53 ± 5 hours in contrast to the significant longer survival (p < 0.05) of the dummy device group (74 ± 6 hours) as demonstrated by the black line in relation to time after hepatectomy (in hours).

Mean operation time (181 ± 24 minutes) and intraoperative blood loss caused by the blood volume remaining in the liver ranged from 300 to 700 mL, did not differ between both groups. Overall postoperative survival of the pigs was 100% after 24 hours. All animals (10/10) (100%) died due to multiple-organ failure and cerebral edema within 63 ± 5 hours after hepatectomy. Survival of the dummy device group (n = 5) was 74 ± 6 hours in contrast to 53 ± 5 hours survival of the control group (n = 5), which was statistically significant (p < 0.05). Kaplan-Meier survival plot of both groups is shown as Figure 1.

### Clinical course of body temperature

**Figure 2:**
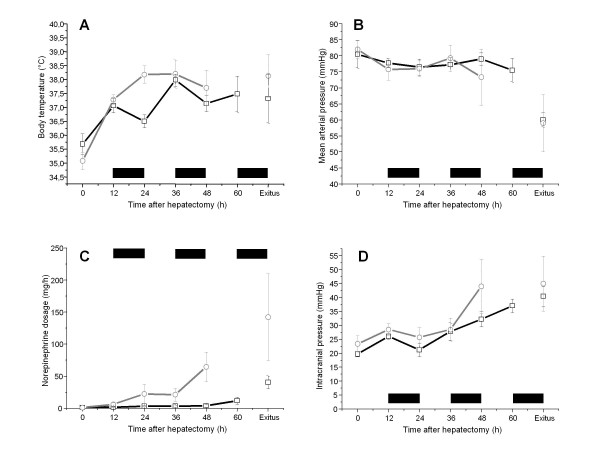
**Profile of body temperature, MAP, norepinephrine concentration and ICP in control and dummy device group**. Chart A demonstrates the clinical course of body temperature (C°) of the control (grey line) and dummy device group (black line). A cyclic decline could be observed in correlation to the connection cycles. Body temperature, 24 hours after hepatectomy, differ statistically significant (p < 0.05) between the groups. Furthermore chart B and C demonstrate more hemodynamic stability of the dummy device (black line) versus the control group (grey line) presented by the nearly identical course of MAP but the lower amount of vasopressor support. ICP values are presented in chart D. Values did not differ statistically significant between the groups over the observed survival. All values are given as mean ± SEM in relation to time after hepatectomy (in hours). Bold marks indicate connection cycles to the dummy device.

After completing surgical procedures, body temperature was as low as 35.8 ± 0.4°C (dummy device group) and 35.7 ± 0.3°C (control group). Pigs were slightly warmed with warming blankets. The further course of body temperature is demonstrated in Figure 2A. Temperature of the control group pigs could be increased to values of 38.2 ± 0.7°C 24 hours after hepatectomy. Body temperature of the dummy device group which received the first connection cycle twelve hours after hepatectomy could not be raised to more than 36.5 ± 0.5°C 24 hours after hepatectomy due to extracorporeal cooling, which was statistically significant (p < 0.05). A cyclic decline in body temperature could be observed in correlation to the connection cycles but no further statistical significance was noticed.

### Cardiopulmonary parameters and ICP monitoring

Ventilation deteriorated progressively during anhepatic coma until death. No significant differences concerning oxygen concentration, maximal airway pressure, positive end expiratory pressure and tidal volume could be detected between both groups. No significant differences were found in hemodynamic parameters between both groups during surgical procedures and further anhepatic coma. The amount of fluid administered and the central venous pressure - positive end-expiratory pressure which was targeted did not differ significantly either between both groups [see additional file 1]. A trend to more hemodynamic stability during anhepaty could be demonstrated by the nearly identical clinical course of MAP but a lower amount of vasopressor support in the dummy device group (Figure 2B and 2C). ICP started at the elevated level of 23 ± 5 mmHg (control group) and 20 ± 2 (dummy device group) caused by the unphysiological supine position. During surgical procedures values did not change. Subsequently values increased up to levels of 45 ± 17 mmHg (control group) and 40 ± 8 (dummy device group) when death occurred, but no statistically significant differences could be observed in the course of ICP (Figure 2D).

### Laboratory analysis

**Figure 3:**
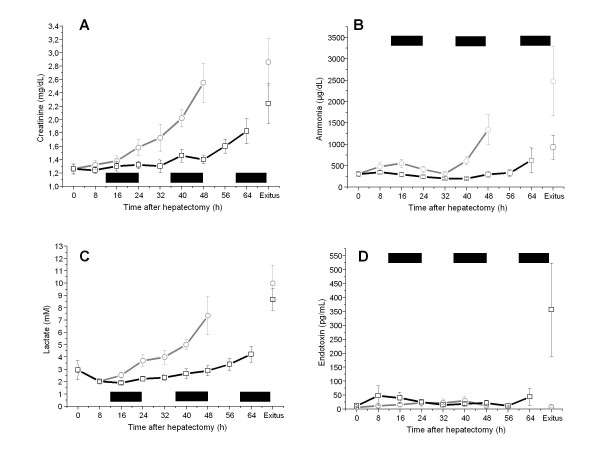
**Profile of creatinine, ammonia, lactate and endotoxin in control and dummy device group**. The chart demonstrates the course of the selected laboratory values creatinine, A, ammonia, B, lactate, C and endotoxin, D of the control (grey line) and dummy device group (black line) animals. A statistical significance (p < 0.05) could be detected for blood lactate starting from 16 hours, serum creatinine from 40 hours and arterial ammonia from 48 hours after hepatectomy. At time of death no differences were noticed for creatinine, ammonia and lactate, but a statistically significant (p < 0.05) rise of endotoxin serum levels indicated severe sepsis in 60% of the animals surviving beyond 60 hours from hepatectomy. Values are given as mean ± SEM in relation to time after hepatectomy (in hours). Bold marks indicate connection cycles to the dummy device.

Laboratory values of all pigs were found to start within the normal range at the time of surgery (table 1). Postoperatively, biochemical parameters as blood count, total plasma protein, albumin, prothrombin time, creatinine, ammonia and endotoxin did not differ significantly. Approximately 6 hours after surgery blood lactate was 2.2 ± 0.3 mmol/L in both study groups representing equal outcome after completing all surgical procedures. The further clinical course of the selected parameters creatinine, ammonia and lactate is shown in Figure 3A-C. Significant (p < 0.05) lower laboratory values of the dummy device group animals were detected starting from 16 hours after hepatectomy for blood lactate (1.9 ± 0.2 vs. 2.5 ± 0.5 mM/L), from 40 hours for serum creatinine (1.5 ± 0.2 vs. 2.0 ± 0.3 mg/dL) and from 48 hours for arterial ammonia (273 ± 122 vs. 1345 ± 700 μg/dL) until death as shown in table 1. At time of death no statistically significance for creatinine, ammonia and lactate values was noticed representing death due to ALF. Endotoxin measurements remained at baseline over the observed survival time in all control animals, but 60% (3/5) of the dummy device pigs showed a significant rise of endotoxin levels (356 ± 373 vs. 9 ± 7 pg/mL, p < 0.05) at time of death (Figure 3D).

**Table 1:** Body temperature and laboratory values of selected biochemical parameters after hepatectomy.

	post-surgery		24 h		48 h		72 h		Exitus	
	Control group	Dummy device	Control group	Dummy device	Control group	Dummy device	Control group	Dummy device	Control group	Dummy device
Temperature (°C)	35.7 ± 0.3	35.8 ± 0.4	38.2 ± 0.7*	36.5 ± 0.5*	37.7 ± 1.3	37.1 ± 0.7	-	37.4 ± 0.4	38.1 ± 1.5	37.3 ± 2.0
Total protein (g/dL)	4.0 ± 0.9	3.9 ± 0.2	5.8 ± 1.0	4.9 ± 0.5	5.1 ± 0.3	5.5 ± 1.0	-	6.3 ± 0.2	4.4 ± 1.7	4.0 ± 1.1
Albumin (g/dL)	2.4 ± 0.6	2.4 ± 0.1	3.3 ± 0.5	2.8 ± 0.3	2.8 ± 0.2	3.1 ± 0.5	-	3.5 ± 0	2.4 ± 1.0	2.2 ± 0.6
PT (%)	83 ± 25	97 ± 18	60 ± 17	41 ± 5	50 ± 17	56 ± 14	-	52 ± 4	43 ± 24	34 ± 11
Creatinine (mg/dL)	1.3 ± 0.2	1.3 ± 0.2	1.6 ± 0.3	1.3 ± 0.1	2.6 ± 0.6*	1.4 ± 0.2*	-	2.1 ± 0.8	2.9 ± 0.8	2.2 ± 0.7
Platelets (10^3^/μL)	240 ± 112	331 ± 99	180 ± 59	215 ± 74	128 ± 79	137 ± 43	-	133 ± 63	90 ± 33	59 ± 29
Haemoglobin (g/dL)	9.5 ± 1.7	9.7 ± 1.7	8.8 ± 1.1	8.5 ± 0.7	6.7 ± 2.0	8.4 ± 1.1	-	8.8 ± 0.2	5.3 ± 1.5	6.5 ± 1.1
Ammonia (μg/dL)	310 ± 98	276 ± 93	411 ± 169	233 ± 65	1345 ± 700*	273 ± 122*	-	307 ± 210	2473 ± 1808	977 ± 499
Bilirubin (mg/dL)	0.2 ± 0.1	0.3 ± 0.2	2.0 ± 0.7	1.6 ± 0.4	2.8 ± 1.8	3.1 ± 0.9	-	3.8 ± 0	2.3 ± 1.9	2.3 ± 0.8
Leukocytes (10^3^/μL)	12 ± 5	17 ± 3	24 ± 15	35 ± 11	23 ± 14	30 ± 11	-	27 ± 14	14 ± 6	9 ± 9
Lactate (mM)	2.9 ± 1.7	2.9 ± 1.7	3.7 ± 1.1*	2.2 ± 0.4*	7.4 ± 3.1*	2.9 ± 0.9*		4.6 ± 3.3	10.0 ± 3.3	8.7 ± 2.0

The course of body temperature and values of selected laboratory parameters after surgery, 24 hours, 48 hours, 72 hours and at time of death. Bold values* indicates a statistically significant difference (p < 0.05) between the control and dummy device group. Values are reported as mean ± SD.

### Autopsy

At autopsy massive ascites (2,000 to 3,000 mL) was found in all animals, no signs of intestinal congestion were noticed and all vascular grafts were found to be regularly patent without either signs of thrombosis or evidence of bleeding from anastomosis side. Kidneys were swollen and showed hemorrhagic infarctions; histological examinations confirmed tubular necrosis. Histological examinations of the brain revealed massive oedema.

## Discussion

Based on preliminary pig studies with a model of total hepatectomy [11] and standardized intensive care therapy [18], we performed the presented study to evaluate a dummy device effects on hemodynamics, body temperature and survival.

Although the used dummy device system was not created as a therapeutic approach, a significant survival benefit of more than 20 hours was noticed. The courses and values of the selected laboratory parameters ammonia, lactate, creatinine or endotoxin which are appropriate to verify different aspects of detoxification were analysed before and after connection to the dummy device. As it was expected, no significant changes of these parameters were observed during or after dummy device connections. Therefore it was demonstrated that these parameters were neither improved nor changed by cyclic application of the dummy device. Our observations were confirmed by the recently published animal study of Ho et al. [25] analysing selective plasma filtration in a porcine model of ALF. A nearly identical experimental device setting (plasma separation with return of the undetoxified plasma) served as a control group in which no detoxification aspect was noticed and survival remained comparable to the sham (diseased) control group.

Body temperature of all pigs was aimed to maintain within a range of 38 ± 0.5°C by warming blankets, but this parameter was significantly affected by the cyclic connection to the extracorporeal device. Moderate hypothermia has already been established as a method for organ and brain protection by reducing oxygen metabolism in severe neurosurgical traumas or systemic disorders such as ALF [26]. Surprisingly the intracranial pressure could not be decreased in our experimental setting. This phenomenon might be associated to the fact that the cyclic hypothermia of 1-1.5°C was not effective for decreasing ICP.

The study might be criticized for the absence of an additionally warmed dummy device group with body temperatures corresponding to those of the presented control group or an additionally cooled control group. But the presented study was not designed to confirm the already experimentally [27] and clinically [28] proven merits of therapeutic hypothermia by cooling down the animals to a predefined temperature. Our objective was to analyse the clinical effects of a dummy device to verify their potential impact on survival. Therefore we could demonstrate that even slight episodes of hypothermia which remained considerably above the required interval for a therapeutical hypothermia do significantly enhance hemodynamics and consecutively anhepatic survival.

Although antibiotic prophylaxis was administered daily, a significant rise of endotoxin levels, paralleled by a leukocyte drop (data not shown) occurred at time of death in 60% of the dummy device group animals surviving beyond 60 hours. This observation confirmed the fact that relevant inflammation mediators were not eliminated by binding to the plasma separation filter. The onset of sepsis destabilized the only just compensated circulation of the organism resulting in a sudden uncontrollable circulation failure, contrary to the more progressive course in multiple-organ failure. It therefore marks definitively an endpoint of anhepatic survival which can hardly be prolonged further. Theoretically this phenomenon could also be associated with hypothermia [29], but it is hardly conceivable that cyclic hypothermia of 1-1.5°C causes a relevant destabilisation of the immune system within this experimental setting.

Standardized intensive care management prolonged anhepatic survival. In combination with slight hypothermia it could be extended up to 88 hours. Therefore the anhepatic porcine model provides a valuable tool for screening functionality and safety of liver support technologies but the efficacy of liver assist device should not be assessed by an anhepatic survival benefit. It should be mentioned that as much as you prolong anhepatic survival the more side-effects like potential transient hypothermia or septical complications will disturb the reproducibility of the model. Significant elimination of hepatotoxic substances or relevant detoxification resulting in increased hemodynamic stability could be better surrogate parameters to verify the effectiveness of any therapeutical approach.

## Conclusions

Episodes of slight hypothermia induced by cyclic connection to the extracorporeal dummy device produced a significant survival benefit of more than 20 hours through organ protection and hemodynamic stabilisation. Sepsis as a severe complication of ALF was only noticed in the dummy device group animals surviving beyond 60 hours which definitively marks the endpoint of survival in the anhepatic porcine model. Animal studies evaluating a liver assist device efforts should especially address the aspect of even slight transient hypothermia induced by the extracorporeal cooling in their experimental setting.

## List of abbreviations

ALF: Acute liver failure; SIRS: Systemic inflammatory response syndrome; ICP: Intracranial pressure; MAP: Mean arterial pressure; SD: standard deviation; SEM: standard error of mean.

## Competing interests

The authors declare that they have no competing interests.

## Authors' contributions

KT, MS, AE, TS, MM, CT carried out the studies. KT, MS, CT designed the study and coordinated the study group. KT, MS, MM, CT drafted the manuscript. AK helped to draft the manuscript and participated in its design. AE, TS carried out the biochemical analysis and helped to draft the manuscript. MS performed the statistical analysis. All authors read and approved the final manuscript.

## Pre-publication history

The pre-publication history for this paper can be accessed here:

http://www.biomedcentral.com/1471-230X/11/79/prepub

## Supplementary Material

Additional file 1 **Fluid management and central venous pressure - positive end-expiratory pressure (PEEP) of the control and dummy device group**. Total amount of fluid administered to the animals subdivided in porcine fresh-frozen plasma (300 mL/unit), porcine erythrocyte (300 mL/unit) and colloidal/crystalloid (500 mL/unit) units, of the control and dummy device group after hepatectomy. All values are given as mean ± SD subsumed in 12 hour time periods in relation to time after hepatectomy. Central venous pressure - positive end-expiratory pressure (PEEP) as a parameter of preload is given to demonstrate adequate preload without dilution effects. Click here for file
