# Supplementary material for: A simple dummy liver assist device prolongs anhepatic survival in a porcine model of total hepatectomy by slight hypothermia
Source: BMC Gastroenterol. 2011 Jul 14;11:79. doi: 10.1186/1471-230X-11-79 (PMC3224123; doi:10.1186/1471-230X-11-79)
Supplement: Additional file 1 — Fluid management and central venous pressure - positive end-expiratory pressure (PEEP) of the control and dummy device group. Total amount of fluid administered to the animals subdivided in porcine fresh-frozen plasma (300 mL/unit), porcine erythrocyte (300 mL/unit) and colloidal/crystalloid (500 mL/unit) units, of the control and dummy device group after hepatectomy. All values are given as mean ± SD subsumed in 12 hour time periods in relation to time after hepatectomy. Central venous pressure - positive end-expiratory pressure (PEEP) as a parameter of preload is given to demonstrate adequate preload without dilution effects. [file 1471-230X-11-79-S1.DOC]

**Additional file 1: Fluid management and central venous pressure - positive end-expiratory pressure (PEEP) of the control and dummy device group.**

| Time after Hepatectomy (h) | Total Volume infused (mL) | | | | | | Central venous pressure  – PEEP  (mmHg) | |
| --- | --- | --- | --- | --- | --- | --- | --- | --- |
|  | Crystall. and colloid. sol.  (500 mL/unit) | | Erythrocyte concentrate  (300 mL/unit) | | Fresh-frozen plasma  (300 mL/unit) | |  |  |
|  | Control Group | Dummy Device | Control Group | Dummy Device | Control Group | Dummy Device | Control Group | Dummy Device |
| 0 - 12 | 3875 ± 854 | 3700 ± 908 | 75 ± 150 | 180 ± 164 | 1200 ± 424 | 1020 ± 268 | 6 ± 3 | 5 ± 3 |
| 12 - 24 | 3000 ± 913 | 2700 ± 1095 | 225 ± 150 | 60 ± 134 | 1350 ± 173 | 1200 ± 0 | 8 ± 3 | 10 ± 3 |
| 24 - 36 | 1875 ± 854 | 2100 ± 224 | 375 ± 150 | 120 ± 268 | 1125 ± 377 | 1080 ± 268 | 9 ± 2 | 9 ± 3 |
| 36 - 48 | 2000 ± 816 | 1900 ± 1084 | 375 ± 150 | 480 ± 342 | 1350 ± 387 | 1320 ± 164 | 9 ± 3 | 10 ± 3 |
| 48 - 60 | 1750 ± 1061 | 1900 ± 1084 | 450 ± 212 | 660 ± 391 | 1050 ± 212 | 1440 ± 329 | 9 ± 2 | 11 ± 2 |
| 60 - 72 | 1500 | 1600 ± 418 | 600 | 540 ± 251 | 600 | 900 ± 300 | 10 ± 1 | 11 ± 1 |
| 72 - 84 |  | 1000 ± 707 |  | 300 ± 424 |  | 1200 ± 0 |  | 13 ± 1 |
| 84 - 96 |  | 1500 |  | 300 |  | 600 |  | 13 |

Total amount of fluid administered to the animals subdivided in porcine fresh-frozen plasma (300 mL/unit), porcine erythrocyte (300 mL/unit) and colloidal/crystalloid (500 mL/unit) units, of the control and dummy device group after hepatectomy. All values are given as mean ± SD subsumed in 12 hour time periods in relation to time after hepatectomy. Central venous pressure - positive end-expiratory pressure (PEEP) as a parameter of preload is given to demonstrate adequate preload without dilution effects.
